# Supplementary material for: Computational exploration of molecular receptive fields in the olfactory bulb reveals a glomerulus-centric chemical map
Source: Sci Rep. 2020 Jan 9;10:77. doi: 10.1038/s41598-019-56863-4 (PMC6952415; doi:10.1038/s41598-019-56863-4)
Supplement: Supplementary file 1 — Supplementary results, figures, and data. [file 41598_2019_56863_MOESM1_ESM.pdf]

Computational exploration of molecular receptive fields in the olfactory bulb reveals a glomerulus-centric chemical map
# – Supplementary information –

Jan Soelter[1], Jan Schumacher[2], Hartwig Spors[2,§], Michael Schmuker[1,3]

1: Neuroinformatics & Theoretical Neuroscience, Freie Universität Berlin, Königin-Luise-Str. 1-3, 14195 Berlin.

2: Max-Planck-Institute for Biophysics, Max-von-Laue-Str. 3, 60438 Frankfurt/Main, Germany

3: School of Computer Science, University of Hertfordshire, Hatfield, Hertfordshire AL10 9AB, United Kingdom.

§: Present address: Dept. of Neuropediatrics, Max-Liebig-University, Giessen, Germany

Corresponding author:
Dr. Michael Schmuker
Reader in Data Science
Biocomputation group
School of Computer Science
University of Hertfordshire
Hatfield, Herts AL10 9AB
United Kingdom
email: m.schmuker@biomachinelearning.net

Replication guide

We provide the complete codebase and data to replicate all figures in this contribution. Jupyter notebooks for replicating figures are provided at https://github.com/Huitzilo/glomcentric_code. The reference release is v2.0, archived at https://doi.org/10.5281/zenodo.1745464. Data can be found at https://doi.org/10.5281/zenodo.1297376 .

Steps to replicate:

1. Download and extract the data (5 GB download, 13 GB extracted).
2. Clone the code repository (will need about 130 MB after replication).
3. Required python packages and versions are listed in soelteretal.yml in the code repository. This file can be imported into Anaconda Python (python 2.7). Set up and activate a virtual environment (or conda environment) with the exact versions provided. We strongly encourage using the exact versions provided, since version conflicts are likely to cause errors that are difficult to detect and diagnose.
4. Launch jupyter notebook in the top-level directory of the code repository.
5. Launch MOB_Fig_decomp_and_spec.ipynb and follow the instructions.

Use the following jupyter notebooks to reproduce each figure:

Figures 1 and 2: MOB_Fig_decomp_and_spec.ipynb

Figure 3: MOB_Fig_SpH.ipynb

Figure 4: MOB_Fig_univar_multivar.ipynb

Figures 5 and 6: MOB_Fig_tunotopy.ipynb

Figure 7: MOB_Fig_chemotopic_clustering.ipynb

Reporting comments, bug reports and other issues is encouraged and should happen via the "issues" functionality in the github repository.

Supplemental results: Single descriptor models

We tested if a single descriptor is sufficient to explain the receptor activation. That is for each descriptor we evaluated different functional relationships between its value and receptor activation:

I. Linear increase of activation with descriptor value (linear regression)

II. Arbitrary (non-parametric) monotonic increase of activation (isotonic regression), e.g. saturated and/or thresholded increase of activation with descriptor value

III. Peak activation at intermediate descriptor value with monotonic decreasing activation both to lower and higher descriptor values (unimodal regression). This could for example be a scenario for chain length dependency.

For each of the three model classes Figure S3a depicts the best model over all descriptors with respect to the coefficient of determination R^2, i.e., the fraction of explained variance. With increasing model complexity more and more of the response variance became explained (Fig. S3b). However, none of the models could correctly capture the response to all odours. This shortcoming was especially apparent when evaluating the predictive power of the models (Fig. S3c). To this end we fitted the models to bootstrap sub-samples of the data. We then compared predictions to observed values for odours excluded in the sub-sample and calculated the coefficient of determination q^2 for unseen odours (see methods). The predictions both included odours with erroneous assigned activation and missed activation. This was reflected by a coefficient of determination q^2 below 0.25.

Supplemental figures

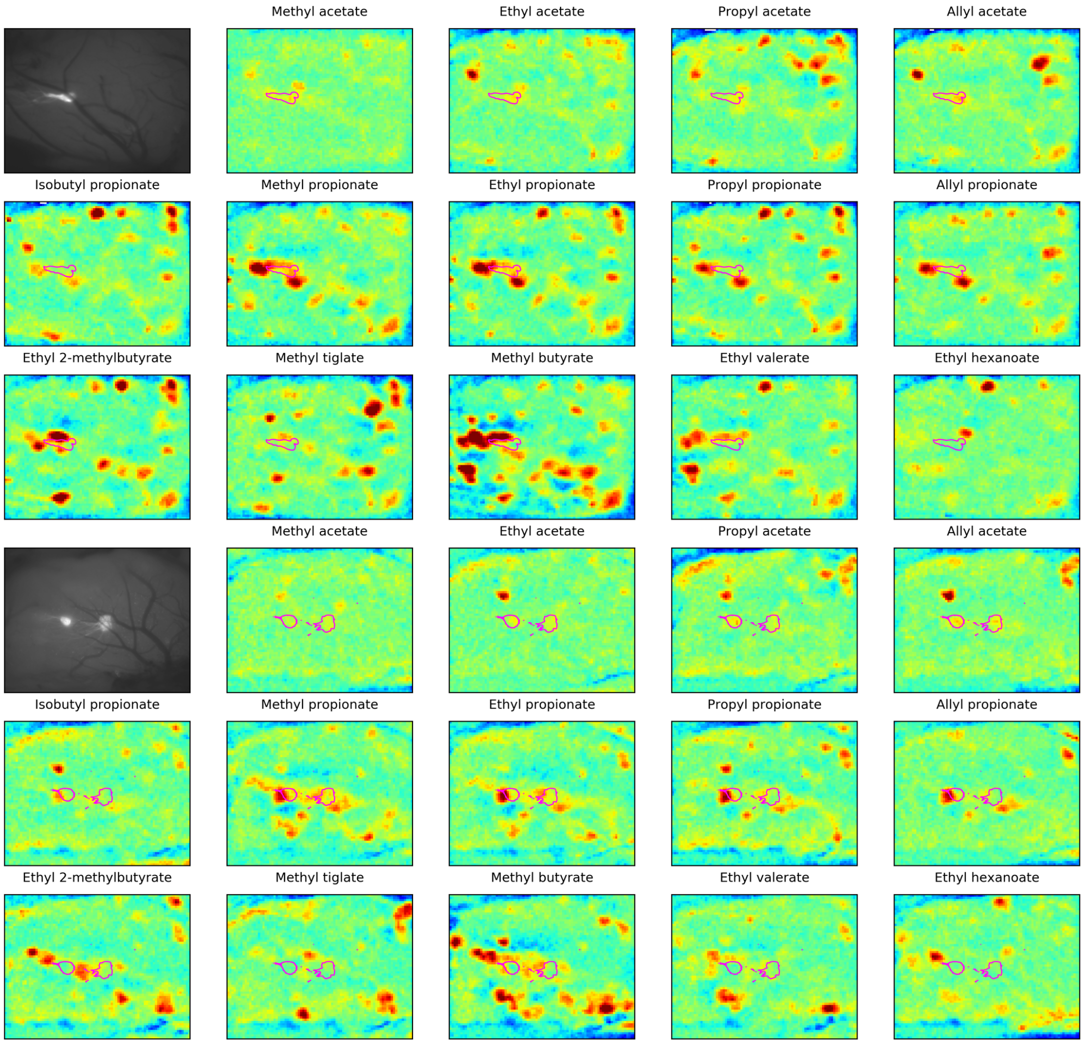

Figure S1: Exemplary Odorant maps. Odorant response in the dOB for a selection of small ester in two different animals. Position of MOR18-2 is marked by red contour lines.

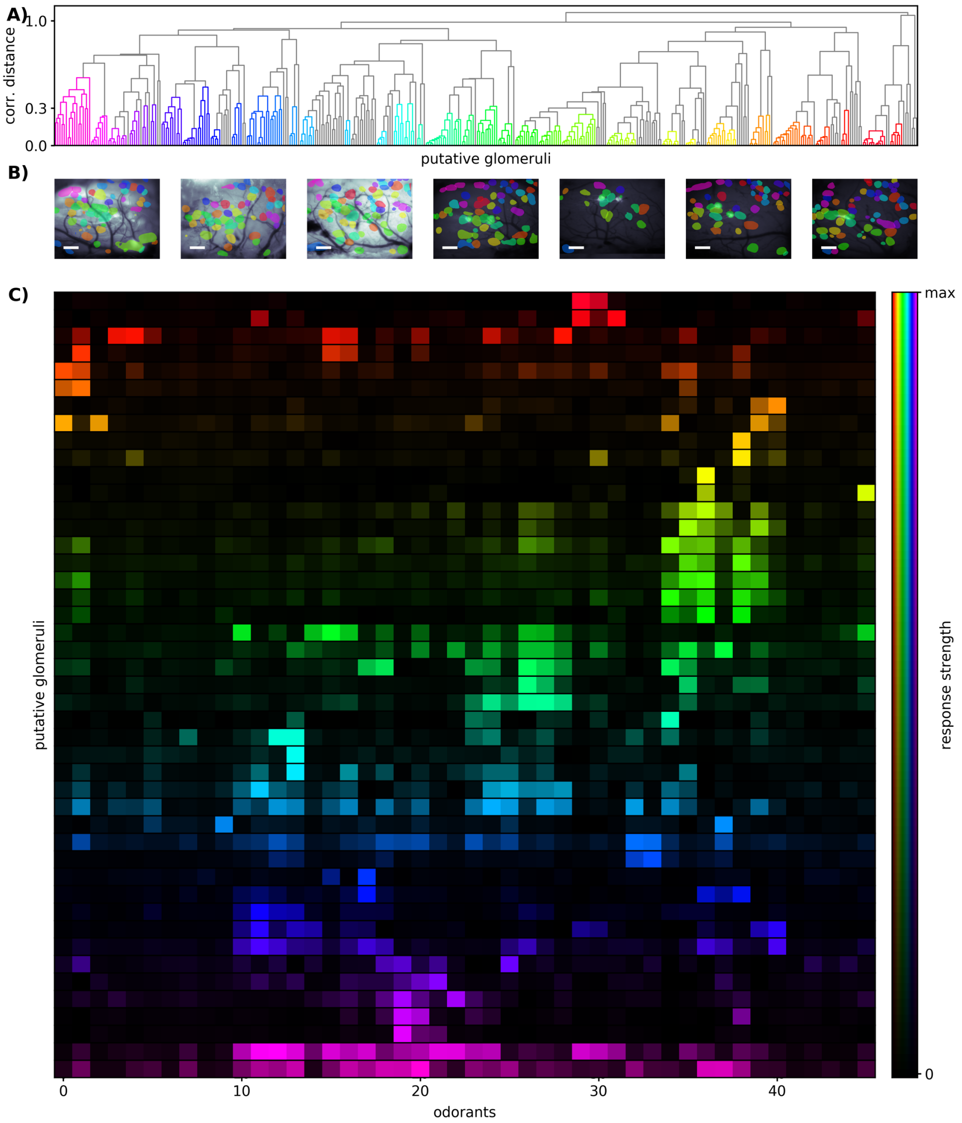

Figure S2: Response Clustering (a) Hierarchical clustering of correlation distance d_r between glomeruli of 7 mice. Length of branches depicts the average correlation distance $\hat{d}_r$ between clades. Properties of coloured clusters are shown in the following panels. (b) Spatial location of cluster members in the OBs of all seven mice. Colours according to cluster colouring in (a). Locations extracted from IOS imaging are overlayed on green images (first 3 animals) respectively GFP images (last 4 animals). (c) Median odour spectra of all glomeruli in each cluster.

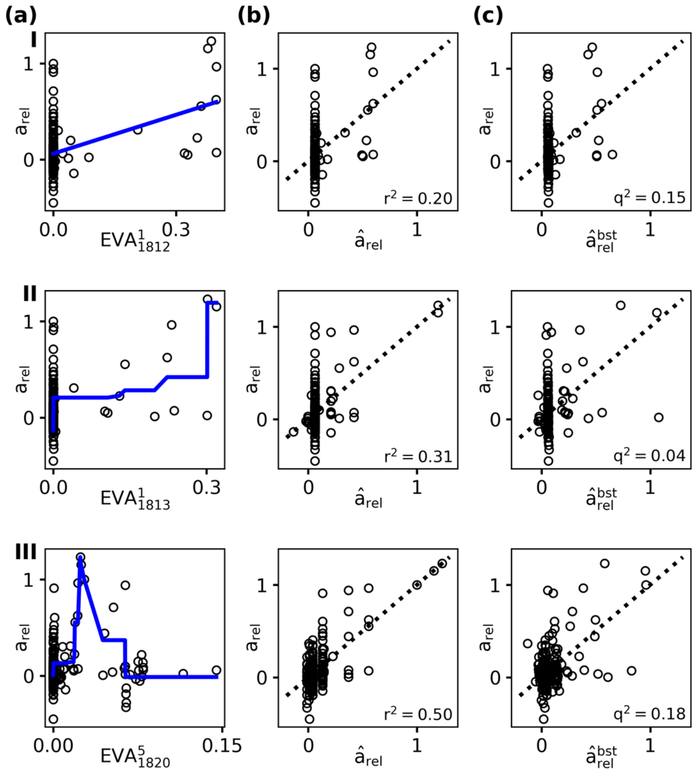

Figure S3: Single-descriptor models. (a) MOR18-2 activation a_{rel} in dependence of descriptor values and corresponding regression models (blue lines): I. linear regression, II. isotonic regression and III. unimodal regression. Each descriptor was chosen to represent the best possible regression model. (b) Observed activations a_{rel} vs. model values $\hat{a}_{rel}$ of the regression models depicted in (a). Resulting fractions of explained variance r^2 is given in the lower right corners. (c) Observed activations a_{rel} vs. bootstrap model predictions $\hat{a}_{rel}^{bst}$ of the same models. Resulting predictive power q^2 is given in the lower right corners.

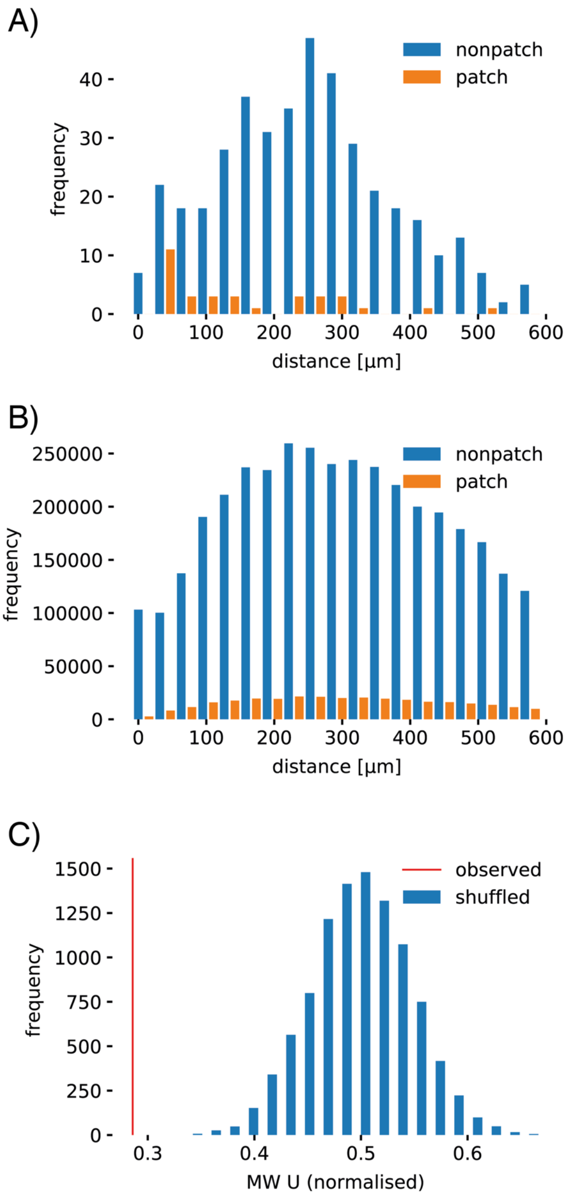

Figure S4: A) Histogram of pairwise distances between glomeruli that belong to the "patch" observed in hierarchical clustering, vs. those that do not belong to the ("non-patch"). Mann-Whitney U statistics yield a normalised U of 0.286 ($p<10^{-4}$). B) Controls with glomerular locations shuffled (10,000 repetitions). C) Histogram of Mann-Whitney U statistics; normalised, i.e. odds of non-patch patch glomeruli being more proximal than patch glomeruli in pairwise comparisons over 10,000 shuffled repetitions. The observed U value is smaller than 100% of the shuffled controls, therefore it can be ruled out that the observed U is obtained by chance (p=0).

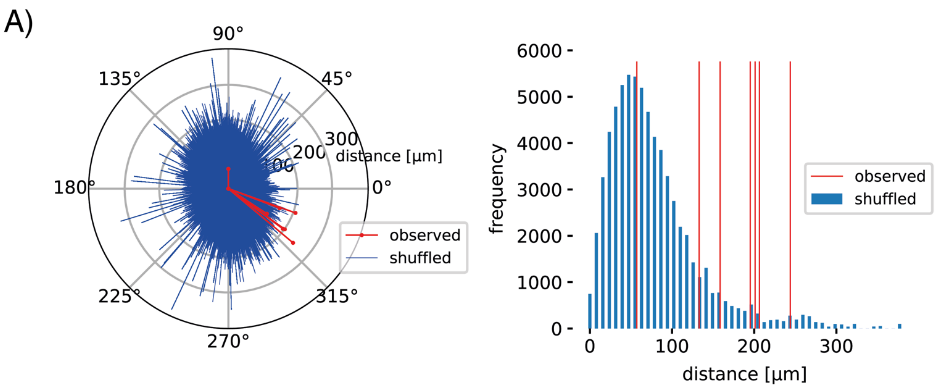

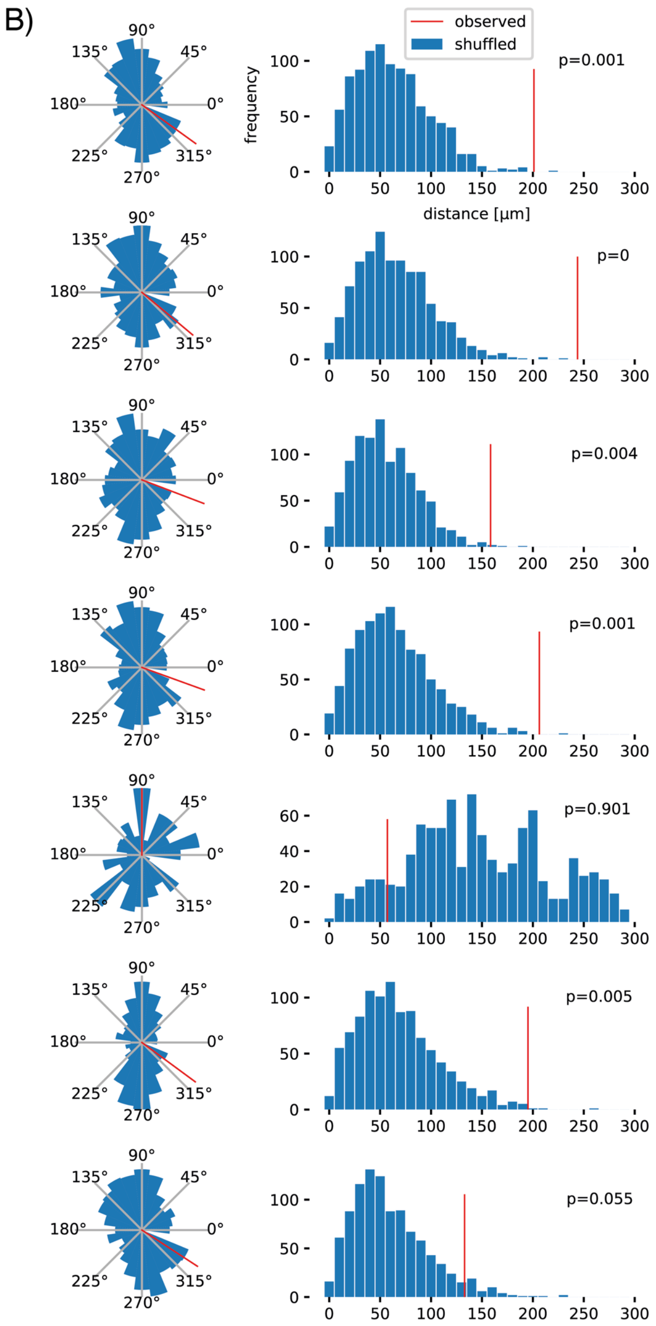

Figure S5: A) Left: Polar plot of observed vectors between centres of mass of red and blue meta-clusters (red), and 10000 shuffled controls (blue). Right: Histogram of distances between the cluster centres as obtained from 10,000 shuffled controls. Red lines indicate observed values. B) Per-animal polar histograms of directions of vectors between centres of mass (left) and distance histograms (right) for 10,000 shuffles. Observed angles and distances are indicated in red.

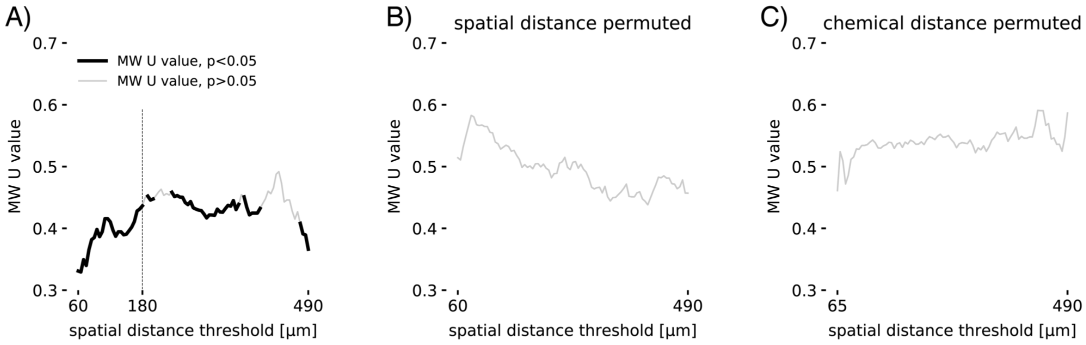

Figure S6: A) Normalised Mann-Whitney U statistics for chemotopic distance of receptive field centres being closer to MOR18-2 within a distance radius or outside. Normalised U values are always below 0.5 and significant ($p<0.05$) for radii up to 180 µm. B) U statistics for shuffled spatial distances, C) shuffled chemical distances. U values for shuffled controls are close to 0.5 and $p>0.05$ for all radii.

Supplementary tables

Supplementary Table ST1: List of all measured odours ranked according to their response at MOR18-2. *P*-value indicates t-test outcome for difference from Argon response, $\log_{10}(p)$ is plotted in Fig. 2.

Index	CAS	Name	MOR18-2 response	*p*-value	**log$_{10}$(*p*)**	in Fig. 3	in Fingerprint/ Fig. 5/6	Num. repl.
1	591-87-7	Allyl acetate	1.14	0.0000	20.40	yes	yes	19
2	141-78-6	Ethyl acetate	1.10	0.0000	23.89	yes	yes	46
3	554-12-1	Methyl propionate	1.00	0.0000	90.28	yes	yes	46
4	109-60-4	Propyl acetate	0.98	0.0000	18.49	yes	yes	17
5	79-20-9	Methyl acetate	0.97	0.0000	13.83	yes	yes	8
6	105-37-3	Ethyl propionate	0.90	0.0000	34.11	yes	yes	18
7	75-07-0	Acetaldehyde	0.75	0.0000	14.44	yes	yes	9
8	2408-20-0	Allyl propionate	0.66	0.0000	16.09	yes	yes	10
9	110-19-0	Isobutyl acetate	0.58	0.0000	19.90	yes	yes	11
10	106-36-5	Propyl propionate	0.55	0.0000	18.28	yes	yes	20
11	123-86-4	Butyl acetate	0.50	0.0000	11.26	yes	yes	13
12	64-19-7	Acetic acid	0.48	1.0000	0.00	yes	-	1
13	5878-19-3	Methoxyacetone	0.47	0.0000	8.16	yes	-	4
14	123-99-9	Azelaic acid	0.44	1.0000	0.00	-	-	1
15	16491-36-4	cis-3-Hexenyl butyrate	0.40	1.0000	0.00	-	-	1
16	109-94-4	Ethyl formate	0.37	0.0000	13.29	yes	-	9
17	108-62-3	Metaldehyde	0.37	1.0000	0.00	-	-	1
18	123-63-7	Paraldehyde	0.31	0.0000	8.80	-	yes	5
19	78-98-8	Pyruvaldehyde	0.30	0.0000	14.65	yes	yes	13
20	140-88-5	Ethyl acrylate	0.29	0.0000	5.90	yes	yes	11
21	19089-92-0	Hexyl trans-2-butenoate	0.28	1.0000	0.00	-	-	1
22	7493-71-2	Allyl tiglate	0.27	0.0000	8.56	yes	yes	17
23	94133-92-3	1-Ethylhexyl tiglate	0.27	0.0000	10.86	-	-	5
24	2349-13-5	Heptyl isobutyrate	0.24	1.0000	0.00	-	-	1
25	540-42-1	Isobutyl propionate	0.23	0.0000	5.63	yes	yes	9
26	108-21-4	Isopropyl acetate	0.23	0.0000	4.60	yes	yes	8
27	4437-51-8	3,4-Hexanedione	0.23	1.0000	0.00	-	-	1
28	41519-18-0	Isoamyl tiglate	0.22	0.0000	6.34	-	-	4
29	7778-87-2	Propyl heptanoate	0.22	1.0000	0.00	-	-	1
30	123-92-2	Isoamyl acetate	0.22	0.0000	5.19	yes	-	13
31	109-21-7	Butyl butyrate	0.21	0.0036	2.45	-	-	7
32	2051-78-7	Allyl butyrate	0.20	0.0040	2.40	yes	-	5

33	105-66-8	Propyl butyrate	0.19	0.0001	3.87	yes	-	8
34	3848-24-6	2,3-Hexanedione	0.17	0.0046	2.34	-	yes	5
35	2639-63-6	Hexyl butyrate	0.17	0.0000	5.34	-	-	5
36	61692-84-0	Isobutyl tiglate	0.17	0.0000	4.46	-	-	5
37	592-84-7	Butyl formate	0.17	1.0000	0.00	-	-	9
38	79-09-4	Propionic acid	0.16	0.0109	1.96	yes	-	8
39	540-18-1	amyl butyrate, mixture of isomers	0.16	0.0001	4.17	-	-	5
40	96-04-8	2,3-Heptanedione	0.16	0.0000	4.93	-	yes	10
41	109-79-5	1-Butanethiol	0.15	1.0000	0.00	-	yes	4
42	52089-55-1	Ethyl (±)-2-hy-droxycaproate	0.15	0.0046	2.34	-	-	4
43	71-23-8	Propanol	0.15	1.0000	0.00	-	-	3
44	503-74-2	Isovaleric acid	0.14	0.0291	1.54	-	-	5
45	637-78-5	Isopropyl propionate	0.14	1.0000	0.00	yes	-	2
46	19788-49-9	Ethyl 2-mercaptopropio-nate	0.14	0.0035	2.45	yes	-	9
47	6342-56-9	Pyruvic aldehyde dime-thyl acetal	0.14	0.0016	2.78	-	-	4
48	107-31-3	Methyl formate	0.13	0.0002	3.80	yes	-	9
49	1733-25-1	Isopropyl tiglate	0.13	1.0000	0.00	yes	-	1
50	623-37-0	3-Hexanol	0.13	1.0000	0.00	yes	-	2
51	431-03-8	2,3-Butanedione	0.12	0.0045	2.35	-	yes	8
52	64-17-5	Ethanol	0.12	1.0000	0.00	-	-	3
53	5910-87-2	trans, trans-2,4-Nonadi-enal	0.12	1.0000	0.00	-	-	1
54	624-24-8	Methyl valerate	0.11	1.0000	0.00	-	-	11
55	591-12-8	alpha-Angelicalactone	0.11	0.0015	2.83	-	-	5
56	140-39-6	p-Tolyl acetate	0.10	1.0000	0.00	yes	-	1
57	638-11-9	Isopropyl butyrate	0.10	0.0074	2.13	yes	-	5
58	107-92-6	Butyric acid	0.09	1.0000	0.00	yes	-	2
59	104-21-2	Anisyl acetate	0.09	1.0000	0.00	-	-	1
60	600-14-6	2,3-Pentanedione	0.09	1.0000	0.00	yes	yes	6
61	106-27-4	Isoamyl butyrate	0.09	0.0200	1.70	-	-	5
62	79-77-6	beta-Ionone	0.09	1.0000	0.00	-	-	2
63	35154-45-1	cis-3-Hexenyl 3-methyl-butanoate	0.09	1.0000	0.00	-	-	1
64	591-80-0	4-Pentenoic acid	0.08	1.0000	0.00	-	-	4
65	623-17-6	Furfuryl acetate	0.08	1.0000	0.00	yes	-	4
66	21835-01-8	3-Ethyl-2-hydroxy-2-cy-clopenten-1-one	0.08	1.0000	0.00	-	-	4
67	591-68-4	Butyl valerate	0.08	1.0000	0.00	-	-	5
68	105-58-8	Diethyl carbonate	0.08	0.0046	2.34	yes	yes	9
69	112-31-2	Decanal	0.08	1.0000	0.00	-	-	1
70	577-16-2	2'-Methylacetophenone	0.08	1.0000	0.00	-	-	1
71	539-82-2	Ethyl valerate	0.08	0.0310	1.51	-	yes	13
72	3268-49-3	3-(Methylthio)propio-naldehyde	0.08	1.0000	0.00	-	-	5

73	1334-82-3	Amyl 2-furoate	0.08	1.0000	0.00	-	-	4
74	2497-18-9	trans-2-Hexenyl acetate	0.08	0.0006	3.21	-	yes	9
75	97-53-0	Eugenol	0.08	1.0000	0.00	-	-	2
76	97-97-2	Chloroacetaldehyde di-methyl acetal	0.07	1.0000	0.00	-	-	4
77	623-19-8	Furfuryl propionate	0.07	1.0000	0.00	-	-	4
78	123-75-1	Pyrrolidine	0.07	1.0000	0.00	-	-	1
79	4455-13-4	Ethyl (methylthio)ace-tate	0.07	1.0000	0.00	-	yes	5
80	116-53-0	(±)-2-Methylbutyric acid	0.07	1.0000	0.00	-	-	4
81	646-07-1	4-Methylvaleric acid	0.07	0.0346	1.46	-	-	4
82	96-48-0	gamma-Butyrolactone	0.07	1.0000	0.00	yes	-	9
83	1759-28-0	4-Methyl-5-vinylthiazole	0.07	1.0000	0.00	-	-	1
84	1122-62-9	2-Acetylpyridine	0.06	1.0000	0.00	-	-	1
85	56-86-0	L-Glutamic acid	0.06	1.0000	0.00	-	-	1
86	2173-56-0	Pentyl valerate	0.05	1.0000	0.00	-	-	5
87	556-82-1	3-Methyl-2-buten-1-ol	0.05	1.0000	0.00	-	-	1
88	142-62-1	Hexanoic acid	0.05	1.0000	0.00	-	-	5
89	112-14-1	Octyl acetate	0.05	1.0000	0.00	-	-	4
90	589-82-2	3-Heptanol	0.05	1.0000	0.00	-	-	4
91	7764-50-3	d-Dihydrocarvone	0.05	1.0000	0.00	-	-	1
92	547-63-7	Methyl isobutyrate	0.05	1.0000	0.00	yes	-	6
93	25680-58-4	2-Ethyl-3-methoxypyra-zine	0.05	1.0000	0.00	-	yes	9
94	97-64-3	Ethyl lactate	0.04	0.0339	1.47	yes	-	7
95	10094-34-5	alpha,alpha-Dime-thylphenethyl butyrate	0.04	1.0000	0.00	-	-	1
96	3391-87-5	(+)-Menthone	0.04	1.0000	0.00	-	-	1
97	106-70-7	Methyl hexanoate	0.04	1.0000	0.00	-	-	5
98	7452-79-1	Ethyl 2-methylbutyrate	0.04	1.0000	0.00	yes	yes	9
99	108-29-2	gamma-Valerolactone	0.04	1.0000	0.00	yes	-	9
100	106-23-0	(±)-Citronellal	0.04	1.0000	0.00	-	yes	6
101	111-11-5	Methyl octanoate	0.03	1.0000	0.00	-	-	10
102	7785-26-4	(-)-alpha-Pinene	0.03	1.0000	0.00	-	-	1
103	103-58-2	3-Phenylpropyl isobutyr-ate	0.03	1.0000	0.00	-	-	1
104	106-24-1	Geraniol	0.03	1.0000	0.00	-	-	4
105	5405-41-4	Ethyl 3-hydroxybutyrate	0.03	0.0352	1.45	-	-	7
106	623-42-7	Methyl butyrate	0.03	1.0000	0.00	yes	yes	12
107	111-88-6	1-Octanethiol	0.03	1.0000	0.00	-	-	1
108	687-47-8	(-)-Ethyl L-Lactate	0.03	1.0000	0.00	-	-	1
109	628-63-7	Pentyl acetate	0.03	0.0231	1.64	yes	yes	9
110	2705-87-5	Allyl cyclohexanepropio-nate	0.03	1.0000	0.00	-	-	9
111	1188-02-9	2-Methylheptanoic acid	0.03	1.0000	0.00	-	-	5
112	5454-19-3	Decyl propionate	0.03	1.0000	0.00	-	-	2
113	7440-37-1	Argon (Control)	0.03	1.0000	0.00	-	yes	44

114	91-22-5	Quinoline	0.03	1.0000	0.00	-	-	1
115	495-40-9	n-Butyrophenone	0.03	1.0000	0.00	-	-	1
116	64-04-0	2-Phenethylamine	0.03	1.0000	0.00	-	-	1
117	1797-74-6	Allyl phenylacetate	0.03	1.0000	0.00	-	-	1
118	95-92-1	Diethyl oxalate	0.03	1.0000	0.00	yes	yes	8
119	695-06-7	gamma-Hexalactone	0.02	1.0000	0.00	yes	-	9
120	5146-66-7	3,7-Dimethyl-2,6-octadi-enenitrile	0.02	1.0000	0.00	-	-	1
121	542-55-2	Isobutyl formate	0.02	1.0000	0.00	yes	yes	13
122	123-11-5	p-Anisaldehyde	0.02	1.0000	0.00	-	-	1
123	98-01-1	2-Furaldehyde	0.02	1.0000	0.00	-	-	5
124	541-85-5	5-Methyl-3-heptanone	0.02	1.0000	0.00	-	-	1
125	141-05-9	Diethyl maleate	0.02	1.0000	0.00	-	-	2
126	104-67-6	gamma-Undecanolac-tone	0.02	1.0000	0.00	-	-	1
127	103-82-2	Phenylacetic acid	0.02	1.0000	0.00	-	-	1
128	10031-92-2	Ethyl 2-nonynoate	0.01	1.0000	0.00	-	-	4
129	79-31-2	Isobutyric acid	0.01	1.0000	0.00	-	-	1
130	89-79-2	(-)-Isopulegol	0.01	1.0000	0.00	-	-	1
131	119-36-8	Methyl salicylate	0.01	1.0000	0.00	-	-	1
132	105-90-8	Geranyl propionate	0.01	1.0000	0.00	-	-	1
133	106-65-0	Dimethyl succinate	0.01	1.0000	0.00	-	-	2
134	106-73-0	Methyl heptanoate	0.01	1.0000	0.00	-	-	5
135	123-68-2	Allyl hexanoate	0.01	1.0000	0.00	-	-	6
136	34047-39-7	4-Methylthio-2-buta-none	0.01	1.0000	0.00	-	-	9
137	6728-26-3	trans-2-Hexenal	0.00	1.0000	0.00	-	-	1
138	288-47-1	Thiazole	0.00	1.0000	0.00	-	-	4
139	540-07-8	Amyl hexanoate	0.00	1.0000	0.00	-	-	1
140	60-12-8	2-Phenylethyl alcohol	0.00	1.0000	0.00	-	yes	7
141	142-92-7	Hexyl acetate	0.00	1.0000	0.00	-	-	9
142	35466-83-2	Allyl methyl carbonate	0.00	0.0327	1.49	yes	yes	9
143	106-32-1	Ethyl caprylate	0.00	0.0246	1.61	-	-	10
144	589-38-8	3-Hexanone	0.00	1.0000	0.00	-	yes	5
145	764-48-7	Ethylene glycol vinyl ether	-0.01	1.0000	0.00	-	-	4
146	93-58-3	Methyl benzoate	-0.01	0.0170	1.77	yes	yes	31
147	5454-28-4	Butyl heptanoate	-0.01	1.0000	0.00	-	-	1
148	67883-79-8	cis-3-Hexenyl tiglate	-0.01	1.0000	0.00	-	-	4
149	40015-15-4	(Methylthio)acetalde-hyde dimethyl acetal	-0.01	1.0000	0.00	-	-	4
150	13679-61-3	Methyl 2-thiofuroate	-0.01	1.0000	0.00	-	-	4
151	89-82-7	(R)-(+)-Pulegone	-0.01	1.0000	0.00	-	yes	5
152	106-22-9	Citronellol	-0.01	1.0000	0.00	-	-	1
153	590-86-3	3-Methylbutyraldehyde	-0.01	0.0220	1.66	-	yes	6
154	107-75-5	3,7-Dimethyl-7-hy-droxyoctanal	-0.02	1.0000	0.00	-	-	1

155	105-68-0	Isoamyl propionate	-0.02	1.0000	0.00	-	-	1
156	7492-70-8	Butyl butyryllactate	-0.02	1.0000	0.00	-	-	1
157	98-86-2	Acetophenone	-0.02	0.0362	1.44	-	-	7
158	123-51-3	3-Methylbutanol	-0.02	1.0000	0.00	-	-	1
159	106-30-9	Ethyl heptanoate	-0.02	1.0000	0.00	-	-	5
160	629-14-1	Ethylene glycol diethyl ether	-0.02	1.0000	0.00	-	-	1
161	138-86-3	Dipentene	-0.02	0.0351	1.45	-	-	3
162	78-70-6	Linalool	-0.03	1.0000	0.00	-	-	4
163	93-89-0	Ethyl benzoate	-0.03	1.0000	0.00	-	-	2
164	122-70-3	2-Phenylethyl propio-nate	-0.03	1.0000	0.00	-	-	1
165	15707-24-1	2,3-Diethylpyrazine	-0.03	1.0000	0.00	-	-	1
166	105-87-3	Geranyl acetate	-0.03	1.0000	0.00	-	-	1
167	105-54-4	Ethyl butyrate	-0.03	1.0000	0.00	yes	-	8
168	6622-76-0	Methyl tiglate	-0.03	0.0060	2.22	-	yes	12
169	15707-23-0	2-Ethyl-3-methylpyrazine	-0.04	1.0000	0.00	-	-	1
170	7540-53-6	Citronellyl valerate	-0.04	0.0070	2.15	-	-	5
171	300-57-2	Allyl benzene	-0.04	1.0000	0.00	-	-	3
172	137-00-8	4-Methyl-5-thiazoleetha-nol	-0.04	1.0000	0.00	-	-	1
173	623-70-1	Ethyl crotonate	-0.04	1.0000	0.00	yes	-	5
174	53448-07-0	trans-2-Undecenal	-0.04	1.0000	0.00	-	-	1
175	67-56-1	Methanol	-0.05	1.0000	0.00	-	-	3
176	624-48-6	Dimethyl maleate	-0.05	1.0000	0.00	yes	-	1
177	108-98-5	Thiophenol	-0.05	1.0000	0.00	-	-	1
178	123-66-0	Ethyl hexanoate	-0.05	0.0045	2.35	-	yes	14
179	1009-14-9	Valerophenone	-0.05	1.0000	0.00	-	-	1
180	123-32-0	2,5-Dimethylpyrazine	-0.05	1.0000	0.00	-	-	2
181	589-98-0	3-Octanol	-0.05	1.0000	0.00	-	-	1
182	97-99-4	Tetrahydrofurfuryl alco-hol	-0.05	0.0142	1.85	-	-	5
183	470-67-7	Cineole	-0.06	0.0286	1.54	-	yes	7
184	100-52-7	Benzaldehyde	-0.06	0.0002	3.66	-	-	6
185	107-87-9	2-Pentanone	-0.06	1.0000	0.00	-	yes	12
186	1128-08-1	Dihydrojasmone	-0.06	1.0000	0.00	-	-	1
187	78-93-3	2-Butanone	-0.07	0.0234	1.63	yes	-	3
188	925-78-0	3-Nonanone	-0.09	0.0184	1.73	-	-	2
189	543-49-7	2-Heptanol	-0.09	0.0049	2.31	-	-	4
190	2396-83-0	Ethyl 3-hexenoate	-0.09	1.0000	0.00	-	-	1
191	51729-83-0	Methyl isopropyl car-bonate	-0.09	0.0000	5.67	-	-	5
192	562-74-3	Terpinen-4-ol	-0.09	1.0000	0.00	-	-	1
193	6976-93-8	Ethylene glycol methyl ether methacrylate	-0.10	0.0001	4.01	-	-	4
194	591-78-6	2-Hexanone	-0.10	0.0002	3.64	-	-	6
195	590-01-2	Butyl propionate	-0.11	0.0021	2.68	yes	yes	5

196	629-19-6	Propyl disulfide	-0.11	0.0067	2.17	-	-	4
197	108-48-5	2,6-Lutidine	-0.11	0.0002	3.74	-	-	4
198	20487-40-5	tert-Butyl propionate	-0.11	0.0076	2.12	-	-	4
199	112-30-1	1-Decanol	-0.11	0.0002	3.72	-	-	4
200	110-93-0	6-Methyl-5-hepten-2-one	-0.13	1.0000	0.00	-	-	1
201	5837-78-5	Ethyl tiglate	-0.13	0.0006	3.19	yes	-	13
202	110-62-3	Valeraldehyde	-0.13	1.0000	0.00	-	-	1
203	623-36-9	2-Methyl-2-pentenal	-0.13	0.0000	5.10	-	yes	24
204	100-66-3	Anisole	-0.13	0.0000	4.66	-	-	18
205	111-13-7	2-Octanone	-0.14	1.0000	0.00	-	-	1
206	110-43-0	2-Heptanone	-0.15	0.0000	5.14	-	-	5
207	124-13-0	Octanal	-0.15	0.0000	5.71	-	-	3
208	25152-84-5	trans, trans-2,4-Decadienal	-0.15	0.0063	2.20	-	-	4
209	68480-28-4	3-Methylbut-2-enyl formate	-0.17	0.0000	6.78	-	-	4
210	19700-21-1	Geosmin	-0.20	1.0000	0.00	-	-	1
211	111-71-7	Heptanal	-0.28	1.0000	0.00	-	-	1
212	121-45-9	Trimethyl phosphite	-0.32	0.0000	10.99	-	-	4
213	66-25-1	Hexanal	-0.33	1.0000	0.00	-	-	1

Supplementary Table ST2: Abbreviations of Descriptor Blocks as used in the manuscript.

**Abbreviation**	**Descriptors**
#FGROUP	FUNCTIONAL GROUP COUNTS
2DAUTO	TWOD AUTOCORRELATIONS
3DMORSE	THREEDMORSE
A_FRAG	ATOMCENTRED FRAGMENTS
BCUT	BURDEN EIGENVALUES DESCRIPTORS
CONST	CONSTITUTIONAL
C_IND	CONNECTIVITY INDICES
EA_IND	EDGE ADJACENCY INDICES
EV_IND	EIGENVALUE INDICES
GEO	GEOMETRICAL
GETAWAY	GETAWAY
MOL_PROP	MOLECULAR PROPERTIES
RAND	RANDIC MOLECULAR PROFILES
RDF	RDF
TOPO	TOPOLOGICAL
WHIM	WHIM
WPATH	WALK PATH COUNTS
